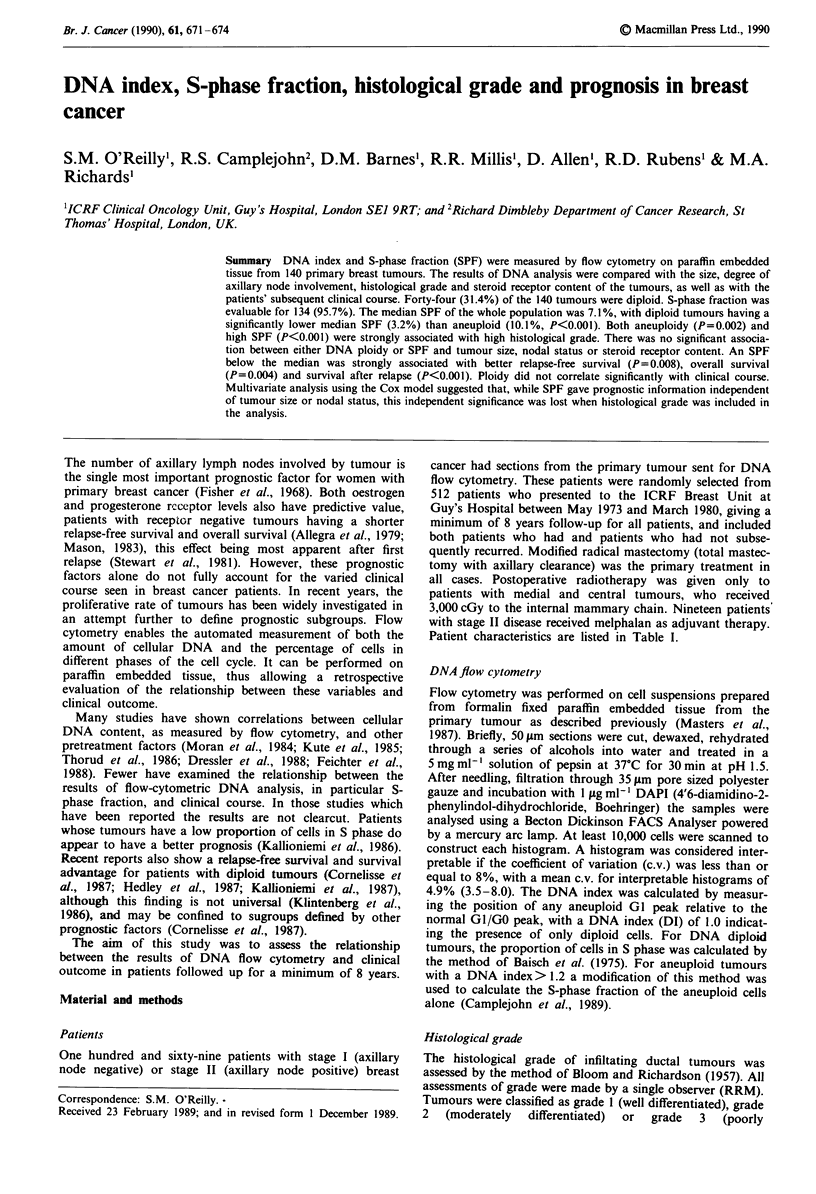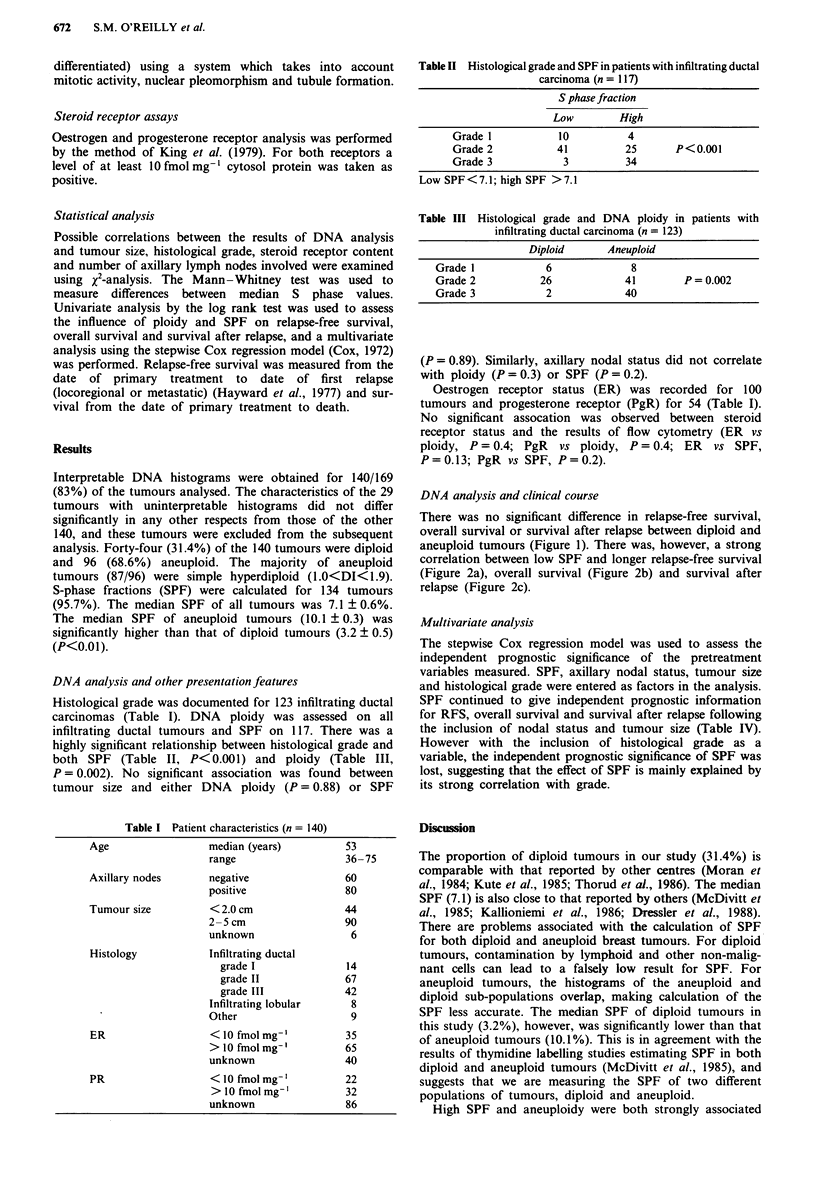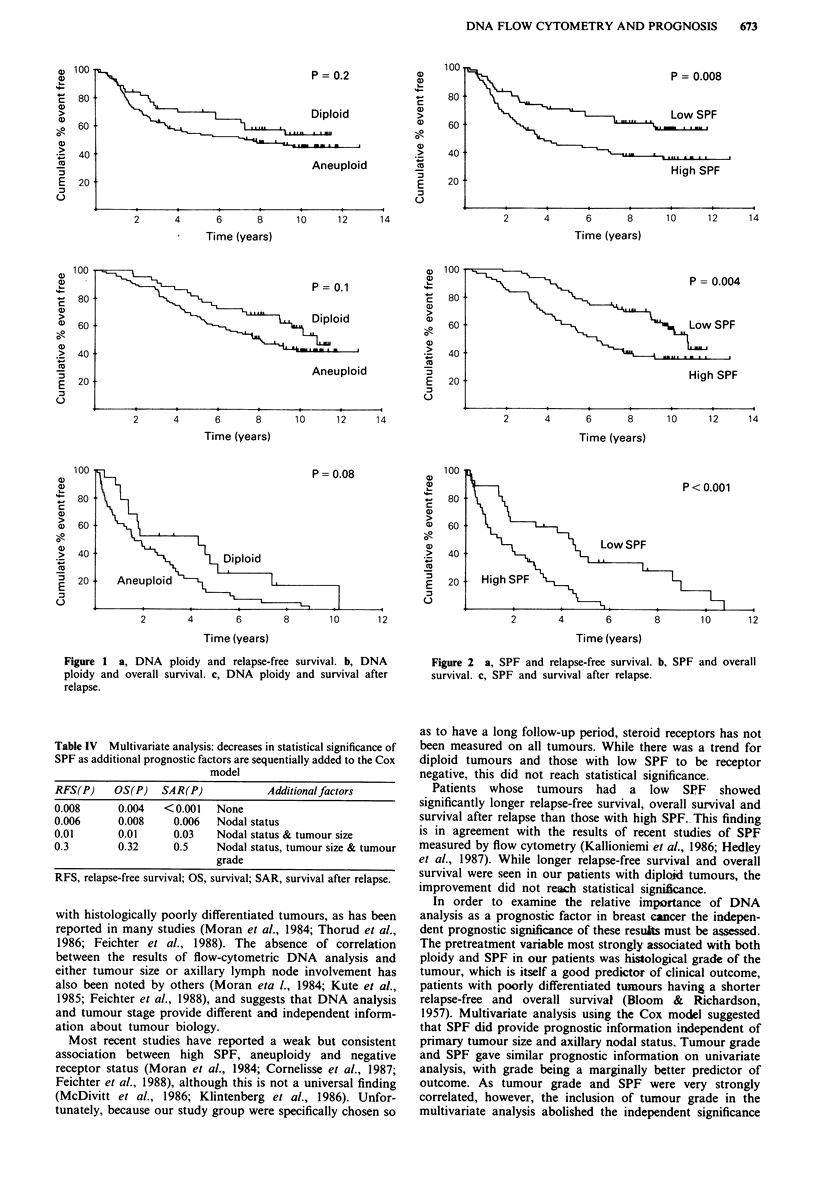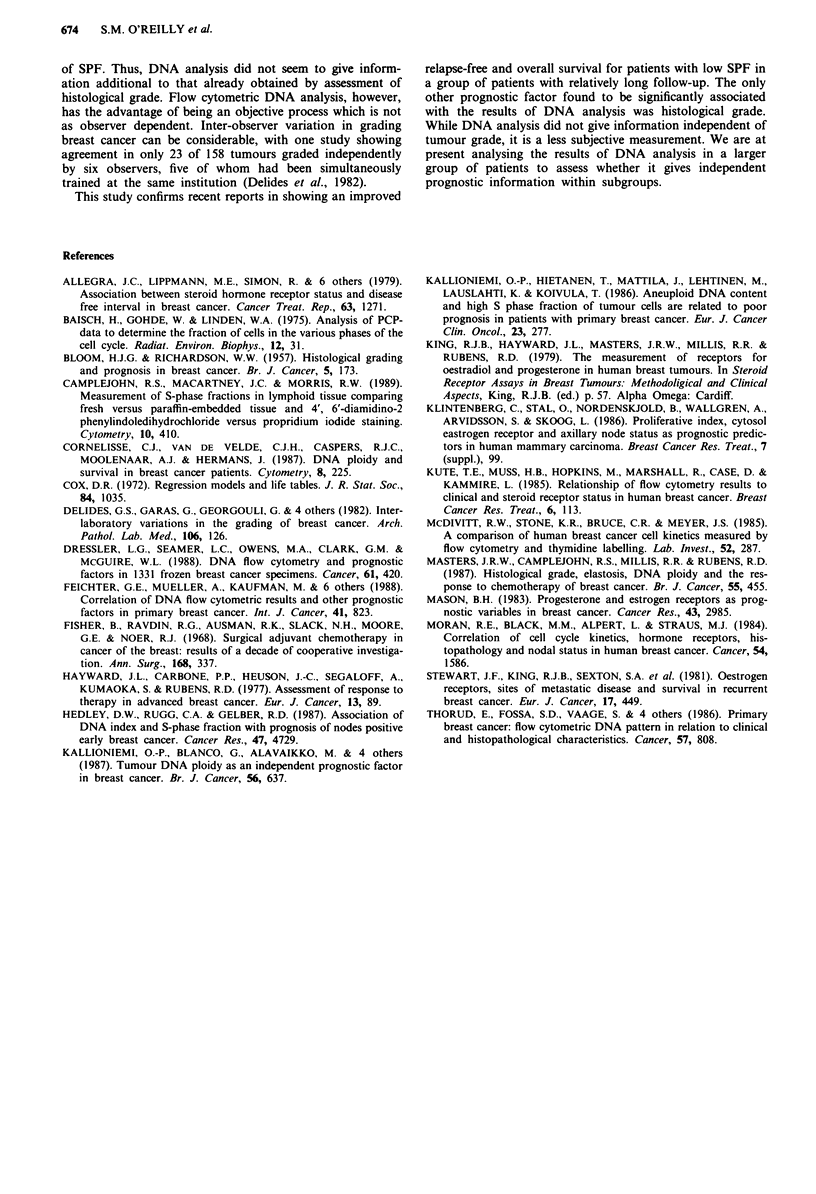# DNA index, S-phase fraction, histological grade and prognosis in breast cancer.

**DOI:** 10.1038/bjc.1990.152

**Published:** 1990-05

**Authors:** S. M. O'Reilly, R. S. Camplejohn, D. M. Barnes, R. R. Millis, D. Allen, R. D. Rubens, M. A. Richards

**Affiliations:** ICRF Clinical Oncology Unit, Guy's Hospital, London, UK.

## Abstract

DNA index and S-phase fraction (SPF) were measured by flow cytometry on paraffin embedded tissue from 140 primary breast tumours. The results of DNA analysis were compared with the size, degree of axillary node involvement, histological grade and steroid receptor content of the tumours, as well as with the patients' subsequent clinical course. Forty-four (31.4%) of the 140 tumours were diploid. S-phase fraction was evaluable for 134 (95.7%). The median SPF of the whole population was 7.1%, with diploid tumours having a significantly lower median SPF (3.2%) than aneuploid (10.1%, P less than 0.001). Both aneuploidy (P = 0.002) and high SPF (P less than 0.001) were strongly associated with high histological grade. There was no significant association between either DNA ploidy or SPF and tumour size, nodal status or steroid receptor content. An SPF below the median was strongly associated with better relapse-free survival (P = 0.008), overall survival (P = 0.004) and survival after relapse (P less than 0.001). Ploidy did not correlate significantly with clinical course. Multivariate analysis using the Cox model suggested that, while SPF gave prognostic information independent of tumour size or nodal status, this independent significance was lost when histological grade was included in the analysis.


Br. J. Cancer (1990), 61, 671-674 © Macmillan Press Ltd., 1990

### DNA index, S-phase fraction, histological grade and prognosis in breast cancer

S.M. O'Reilly[1], R.S. Camplejohn[2], D.M. Barnes[1], R.R. Millis[1], D. Allen[1], R.D. Rubens[1] & M.A. Richards[1]

[1]*ICRF Clinical Oncology Unit, Guy's Hospital, London SE1 9RT; and* [2]*Richard Dimbleby Department of Cancer Research, St Thomas' Hospital, London, UK.*

**Summary** DNA index and S-phase fraction (SPF) were measured by flow cytometry on paraffin embedded tissue from 140 primary breast tumours. The results of DNA analysis were compared with the size, degree of axillary node involvement, histological grade and steroid receptor content of the tumours, as well as with the patients' subsequent clinical course. Forty-four (31.4%) of the 140 tumours were diploid. S-phase fraction was evaluable for 134 (95.7%). The median SPF of the whole population was 7.1%, with diploid tumours having a significantly lower median SPF (3.2%) than aneuploid (10.1%, $P<0.001$). Both aneuploidy ($P=0.002$) and high SPF ($P<0.001$) were strongly associated with high histological grade. There was no significant association between either DNA ploidy or SPF and tumour size, nodal status or steroid receptor content. An SPF below the median was strongly associated with better relapse-free survival ($P=0.008$), overall survival ($P=0.004$) and survival after relapse ($P<0.001$). Ploidy did not correlate significantly with clinical course. Multivariate analysis using the Cox model suggested that, while SPF gave prognostic information independent of tumour size or nodal status, this independent significance was lost when histological grade was included in the analysis.

The number of axillary lymph nodes involved by tumour is the single most important prognostic factor for women with primary breast cancer (Fisher *et al.*, 1968). Both oestrogen and progesterone receptor levels also have predictive value, patients with receptor negative tumours having a shorter relapse-free survival and overall survival (Allegra *et al.*, 1979; Mason, 1983), this effect being most apparent after first relapse (Stewart *et al.*, 1981). However, these prognostic factors alone do not fully account for the varied clinical course seen in breast cancer patients. In recent years, the proliferative rate of tumours has been widely investigated in an attempt further to define prognostic subgroups. Flow cytometry enables the automated measurement of both the amount of cellular DNA and the percentage of cells in different phases of the cell cycle. It can be performed on paraffin embedded tissue, thus allowing a retrospective evaluation of the relationship between these variables and clinical outcome.

Many studies have shown correlations between cellular DNA content, as measured by flow cytometry, and other pretreatment factors (Moran *et al.*, 1984; Kute *et al.*, 1985; Thorud *et al.*, 1986; Dressler *et al.*, 1988; Feichter *et al.*, 1988). Fewer have examined the relationship between the results of flow-cytometric DNA analysis, in particular S-phase fraction, and clinical course. In those studies which have been reported the results are not clearcut. Patients whose tumours have a low proportion of cells in S phase do appear to have a better prognosis (Kallioniemi *et al.*, 1986). Recent reports also show a relapse-free survival and survival advantage for patients with diploid tumours (Cornelisse *et al.*, 1987; Hedley *et al.*, 1987; Kallioniemi *et al.*, 1987), although this finding is not universal (Klintenberg *et al.*, 1986), and may be confined to sugroups defined by other prognostic factors (Cornelisse *et al.*, 1987).

The aim of this study was to assess the relationship between the results of DNA flow cytometry and clinical outcome in patients followed up for a minimum of 8 years.

### Material and methods

### Patients

One hundred and sixty-nine patients with stage I (axillary node negative) or stage II (axillary node positive) breast cancer had sections from the primary tumour sent for DNA flow cytometry. These patients were randomly selected from 512 patients who presented to the ICRF Breast Unit at Guy's Hospital between May 1973 and March 1980, giving a minimum of 8 years follow-up for all patients, and included both patients who had and patients who had not subsequently recurred. Modified radical mastectomy (total mastectomy with axillary clearance) was the primary treatment in all cases. Postoperative radiotherapy was given only to patients with medial and central tumours, who received 3,000 cGy to the internal mammary chain. Nineteen patients with stage II disease received melphalan as adjuvant therapy. Patient characteristics are listed in Table I.

### DNA flow cytometry

Flow cytometry was performed on cell suspensions prepared from formalin fixed paraffin embedded tissue from the primary tumour as described previously (Masters *et al.*, 1987). Briefly, 50 μm sections were cut, dewaxed, rehydrated through a series of alcohols into water and treated in a 5 mg ml$^{-1}$ solution of pepsin at 37°C for 30 min at pH 1.5. After needling, filtration through 35 μm pore sized polyester gauze and incubation with 1 μg ml$^{-1}$ DAPI (4'6-diamidino-2-phenylindol-dihydrochloride, Boehringer) the samples were analysed using a Becton Dickinson FACS Analyser powered by a mercury arc lamp. At least 10,000 cells were scanned to construct each histogram. A histogram was considered interpretable if the coefficient of variation (c.v.) was less than or equal to 8%, with a mean c.v. for interpretable histograms of 4.9% (3.5-8.0). The DNA index was calculated by measuring the position of any aneuploid G1 peak relative to the normal G1/G0 peak, with a DNA index (DI) of 1.0 indicating the presence of only diploid cells. For DNA diploid tumours, the proportion of cells in S phase was calculated by the method of Baisch *et al.* (1975). For aneuploid tumours with a DNA index > 1.2 a modification of this method was used to calculate the S-phase fraction of the aneuploid cells alone (Camplejohn *et al.*, 1989).

### Histological grade

The histological grade of infiltating ductal tumours was assessed by the method of Bloom and Richardson (1957). All assessments of grade were made by a single observer (RRM). Tumours were classified as grade 1 (well differentiated), grade 2 (moderately differentiated) or grade 3 (poorly

Correspondence: S.M. O'Reilly.
Received 23 February 1989; and in revised form 1 December 1989.

differentiated) using a system which takes into account mitotic activity, nuclear pleomorphism and tubule formation.

*Steroid receptor assays*

Oestrogen and progesterone receptor analysis was performed by the method of King *et al.* (1979). For both receptors a level of at least 10 fmol mg$^{-1}$ cytosol protein was taken as positive.

*Statistical analysis*

Possible correlations between the results of DNA analysis and tumour size, histological grade, steroid receptor content and number of axillary lymph nodes involved were examined using $\chi^2$-analysis. The Mann–Whitney test was used to measure differences between median S phase values. Univariate analysis by the log rank test was used to assess the influence of ploidy and SPF on relapse-free survival, overall survival and survival after relapse, and a multivariate analysis using the stepwise Cox regression model (Cox, 1972) was performed. Relapse-free survival was measured from the date of primary treatment to date of first relapse (locoregional or metastatic) (Hayward *et al.*, 1977) and survival from the date of primary treatment to death.

### Results

Interpretable DNA histograms were obtained for 140/169 (83%) of the tumours analysed. The characteristics of the 29 tumours with uninterpretable histograms did not differ significantly in any other respects from those of the other 140, and these tumours were excluded from the subsequent analysis. Forty-four (31.4%) of the 140 tumours were diploid and 96 (68.6%) aneuploid. The majority of aneuploid tumours (87/96) were simple hyperdiploid (1.0<DI<1.9). S-phase fractions (SPF) were calculated for 134 tumours (95.7%). The median SPF of all tumours was 7.1 ± 0.6%. The median SPF of aneuploid tumours (10.1 ± 0.3) was significantly higher than that of diploid tumours (3.2 ± 0.5) ($P$<0.01).

*DNA analysis and other presentation features*

Histological grade was documented for 123 infiltrating ductal carcinomas (Table I). DNA ploidy was assessed on all infiltrating ductal tumours and SPF on 117. There was a highly significant relationship between histological grade and both SPF (Table II, $P$<0.001) and ploidy (Table III, $P$ = 0.002). No significant association was found between tumour size and either DNA ploidy ($P$ = 0.88) or SPF ($P$ = 0.89). Similarly, axillary nodal status did not correlate with ploidy ($P$ = 0.3) or SPF ($P$ = 0.2).

Oestrogen receptor status (ER) was recorded for 100 tumours and progesterone receptor (PgR) for 54 (Table I). No significant assocation was observed between steroid receptor status and the results of flow cytometry (ER *vs* ploidy, $P$ = 0.4; PgR *vs* ploidy, $P$ = 0.4; ER *vs* SPF, $P$ = 0.13; PgR *vs* SPF, $P$ = 0.2).

**Table I** Patient characteristics ($n$ = 140)

| | | |
|---|---|---|
| Age | median (years) | 53 |
| | range | 36–75 |
| Axillary nodes | negative | 60 |
| | positive | 80 |
| Tumour size | <2.0 cm | 44 |
| | 2–5 cm | 90 |
| | unknown | 6 |
| Histology | Infiltrating ductal | |
| | grade I | 14 |
| | grade II | 67 |
| | grade III | 42 |
| | Infiltrating lobular | 8 |
| | Other | 9 |
| ER | < 10 fmol mg$^{-1}$ | 35 |
| | > 10 fmol mg$^{-1}$ | 65 |
| | unknown | 40 |
| PR | < 10 fmol mg$^{-1}$ | 22 |
| | > 10 fmol mg$^{-1}$ | 32 |
| | unknown | 86 |

**Table II** Histological grade and SPF in patients with infiltrating ductal carcinoma ($n$ = 117)

| | *S phase fraction* | | |
|---|---|---|---|
| | *Low* | *High* | |
| Grade 1 | 10 | 4 | |
| Grade 2 | 41 | 25 | $P$<0.001 |
| Grade 3 | 3 | 34 | |

Low SPF<7.1; high SPF >7.1

**Table III** Histological grade and DNA ploidy in patients with infiltrating ductal carcinoma ($n$ = 123)

| | *Diploid* | *Aneuploid* | |
|---|---|---|---|
| Grade 1 | 6 | 8 | |
| Grade 2 | 26 | 41 | $P$ = 0.002 |
| Grade 3 | 2 | 40 | |

*DNA analysis and clinical course*

There was no significant difference in relapse-free survival, overall survival or survival after relapse between diploid and aneuploid tumours (Figure 1). There was, however, a strong correlation between low SPF and longer relapse-free survival (Figure 2a), overall survival (Figure 2b) and survival after relapse (Figure 2c).

*Multivariate analysis*

The stepwise Cox regression model was used to assess the independent prognostic significance of the pretreatment variables measured. SPF, axillary nodal status, tumour size and histological grade were entered as factors in the analysis. SPF continued to give independent prognostic information for RFS, overall survival and survival after relapse following the inclusion of nodal status and tumour size (Table IV). However with the inclusion of histological grade as a variable, the independent prognostic significance of SPF was lost, suggesting that the effect of SPF is mainly explained by its strong correlation with grade.

### Discussion

The proportion of diploid tumours in our study (31.4%) is comparable with that reported by other centres (Moran *et al.*, 1984; Kute *et al.*, 1985; Thorud *et al.*, 1986). The median SPF (7.1) is also close to that reported by others (McDivitt *et al.*, 1985; Kallioniemi *et al.*, 1986; Dressler *et al.*, 1988). There are problems associated with the calculation of SPF for both diploid and aneuploid breast tumours. For diploid tumours, contamination by lymphoid and other non-malignant cells can lead to a falsely low result for SPF. For aneuploid tumours, the histograms of the aneuploid and diploid sub-populations overlap, making calculation of the SPF less accurate. The median SPF of diploid tumours in this study (3.2%), however, was significantly lower than that of aneuploid tumours (10.1%). This is in agreement with the results of thymidine labelling studies estimating SPF in both diploid and aneuploid tumours (McDivitt *et al.*, 1985), and suggests that we are measuring the SPF of two different populations of tumours, diploid and aneuploid.

High SPF and aneuploidy were both strongly associated

**Figure 1** a, DNA ploidy and relapse-free survival. b, DNA ploidy and overall survival. c, DNA ploidy and survival after relapse.

**Figure 2** a, SPF and relapse-free survival. b, SPF and overall survival. c, SPF and survival after relapse.

**Table IV** Multivariate analysis: decreases in statistical significance of SPF as additional prognostic factors are sequentially added to the Cox model

| *RFS(P)* | *OS(P)* | *SAR(P)* | *Additional factors* |
|---|---|---|---|
| 0.008 | 0.004 | <0.001 | None |
| 0.006 | 0.008 | 0.006 | Nodal status |
| 0.01 | 0.01 | 0.03 | Nodal status & tumour size |
| 0.3 | 0.32 | 0.5 | Nodal status, tumour size & tumour grade |

RFS, relapse-free survival; OS, survival; SAR, survival after relapse.

with histologically poorly differentiated tumours, as has been reported in many studies (Moran *et al.*, 1984; Thorud *et al.*, 1986; Feichter *et al.*, 1988). The absence of correlation between the results of flow-cytometric DNA analysis and either tumour size or axillary lymph node involvement has also been noted by others (Moran *eta l.*, 1984; Kute *et al.*, 1985; Feichter *et al.*, 1988), and suggests that DNA analysis and tumour stage provide different and independent information about tumour biology.

Most recent studies have reported a weak but consistent association between high SPF, aneuploidy and negative receptor status (Moran *et al.*, 1984; Cornelisse *et al.*, 1987; Feichter *et al.*, 1988), although this is not a universal finding (McDivitt *et al.*, 1986; Klintenberg *et al.*, 1986). Unfortunately, because our study group were specifically chosen so as to have a long follow-up period, steroid receptors has not been measured on all tumours. While there was a trend for diploid tumours and those with low SPF to be receptor negative, this did not reach statistical significance.

Patients whose tumours had a low SPF showed significantly longer relapse-free survival, overall survival and survival after relapse than those with high SPF. This finding is in agreement with the results of recent studies of SPF measured by flow cytometry (Kallioniemi *et al.*, 1986; Hedley *et al.*, 1987). While longer relapse-free survival and overall survival were seen in our patients with diploid tumours, the improvement did not reach statistical significance.

In order to examine the relative importance of DNA analysis as a prognostic factor in breast cancer the independent prognostic significance of these results must be assessed. The pretreatment variable most strongly associated with both ploidy and SPF in our patients was histological grade of the tumour, which is itself a good predictor of clinical outcome, patients with poorly differentiated tumours having a shorter relapse-free and overall survival (Bloom & Richardson, 1957). Multivariate analysis using the Cox model suggested that SPF did provide prognostic information independent of primary tumour size and axillary nodal status. Tumour grade and SPF gave similar prognostic information on univariate analysis, with grade being a marginally better predictor of outcome. As tumour grade and SPF were very strongly correlated, however, the inclusion of tumour grade in the multivariate analysis abolished the independent significance of SPF. Thus, DNA analysis did not seem to give information additional to that already obtained by assessment of histological grade. Flow cytometric DNA analysis, however, has the advantage of being an objective process which is not as observer dependent. Inter-observer variation in grading breast cancer can be considerable, with one study showing agreement in only 23 of 158 tumours graded independently by six observers, five of whom had been simultaneously trained at the same institution (Delides *et al.*, 1982).

This study confirms recent reports in showing an improved relapse-free and overall survival for patients with low SPF in a group of patients with relatively long follow-up. The only other prognostic factor found to be significantly associated with the results of DNA analysis was histological grade. While DNA analysis did not give information independent of tumour grade, it is a less subjective measurement. We are at present analysing the results of DNA analysis in a larger group of patients to assess whether it gives independent prognostic information within subgroups.

### References

ALLEGRA, J.C., LIPPMANN, M.E., SIMON, R. & 6 others (1979). Association between steroid hormone receptor status and disease free interval in breast cancer. *Cancer Treat. Rep.*, **63**, 1271.

BAISCH, H., GOHDE, W. & LINDEN, W.A. (1975). Analysis of PCP-data to determine the fraction of cells in the various phases of the cell cycle. *Radiat. Environ. Biophys.*, **12**, 31.

BLOOM, H.J.G. & RICHARDSON, W.W. (1957). Histological grading and prognosis in breast cancer. *Br. J. Cancer*, **5**, 173.

CAMPLEJOHN, R.S., MACARTNEY, J.C. & MORRIS, R.W. (1989). Measurement of S-phase fractions in lymphoid tissue comparing fresh versus paraffin-embedded tissue and 4′, 6′-diamidino-2 phenylindoledihydrochloride versus propridium iodide staining. *Cytometry*, **10**, 410.

CORNELISSE, C.J., VAN DE VELDE, C.J.H., CASPERS, R.J.C., MOOLENAAR, A.J. & HERMANS, J. (1987). DNA ploidy and survival in breast cancer patients. *Cytometry*, **8**, 225.

COX, D.R. (1972). Regression models and life tables. *J. R. Stat. Soc.*, **84**, 1035.

DELIDES, G.S., GARAS, G., GEORGOULI, G. & 4 others (1982). Inter-laboratory variations in the grading of breast cancer. *Arch. Pathol. Lab. Med.*, **106**, 126.

DRESSLER, L.G., SEAMER, L.C., OWENS, M.A., CLARK, G.M. & McGUIRE, W.L. (1988). DNA flow cytometry and prognostic factors in 1331 frozen breast cancer specimens. *Cancer*, **61**, 420.

FEICHTER, G.E., MUELLER, A., KAUFMAN, M. & 6 others (1988). Correlation of DNA flow cytometric results and other prognostic factors in primary breast cancer. *Int. J. Cancer*, **41**, 823.

FISHER, B., RAVDIN, R.G., AUSMAN, R.K., SLACK, N.H., MOORE, G.E. & NOER, R.J. (1968). Surgical adjuvant chemotherapy in cancer of the breast: results of a decade of cooperative investigation. *Ann. Surg.*, **168**, 337.

HAYWARD, J.L., CARBONE, P.P., HEUSON, J.-C., SEGALOFF, A., KUMAOKA, S. & RUBENS, R.D. (1977). Assessment of response to therapy in advanced breast cancer. *Eur. J. Cancer*, **13**, 89.

HEDLEY, D.W., RUGG, C.A. & GELBER, R.D. (1987). Association of DNA index and S-phase fraction with prognosis of nodes positive early breast cancer. *Cancer Res.*, **47**, 4729.

KALLIONIEMI, O.-P., BLANCO, G., ALAVAIKKO, M. & 4 others (1987). Tumour DNA ploidy as an independent prognostic factor in breast cancer. *Br. J. Cancer*, **56**, 637.

KALLIONIEMI, O.-P., HIETANEN, T., MATTILA, J., LEHTINEN, M., LAUSLAHTI, K. & KOIVULA, T. (1986). Aneuploid DNA content and high S phase fraction of tumour cells are related to poor prognosis in patients with primary breast cancer. *Eur. J. Cancer Clin. Oncol.*, **23**, 277.

KING, R.J.B., HAYWARD, J.L., MASTERS, J.R.W., MILLIS, R.R. & RUBENS, R.D. (1979). The measurement of receptors for oestradiol and progesterone in human breast tumours. In *Steroid Receptor Assays in Breast Tumours: Methodoligical and Clinical Aspects*, King, R.J.B. (ed.) p. 57. Alpha Omega: Cardiff.

KLINTENBERG, C., STAL, O., NORDENSKJOLD, B., WALLGREN, A., ARVIDSSON, S. & SKOOG, L. (1986). Proliferative index, cytosol eastrogen receptor and axillary node status as prognostic predictors in human mammary carcinoma. *Breast Cancer Res. Treat.*, **7** (suppl.), 99.

KUTE, T.E., MUSS, H.B., HOPKINS, M., MARSHALL, R., CASE, D. & KAMMIRE, L. (1985). Relationship of flow cytometry results to clinical and steroid receptor status in human breast cancer. *Breast Cancer Res. Treat.*, **6**, 113.

McDIVITT, R.W., STONE, K.R., BRUCE, C.R. & MEYER, J.S. (1985). A comparison of human breast cancer cell kinetics measured by flow cytometry and thymidine labelling. *Lab. Invest.*, **52**, 287.

MASTERS, J.R.W., CAMPLEJOHN, R.S., MILLIS, R.R. & RUBENS, R.D. (1987). Histological grade, elastosis, DNA ploidy and the response to chemotherapy of breast cancer. *Br. J. Cancer*, **55**, 455.

MASON, B.H. (1983). Progesterone and estrogen receptors as prognostic variables in breast cancer. *Cancer Res.*, **43**, 2985.

MORAN, R.E., BLACK, M.M., ALPERT, L. & STRAUS, M.J. (1984). Correlation of cell cycle kinetics, hormone receptors, histopathology and nodal status in human breast cancer. *Cancer*, **54**, 1586.

STEWART, J.F., KING, R.J.B., SEXTON, S.A. *et al.* (1981). Oestrogen receptors, sites of metastatic disease and survival in recurrent breast cancer. *Eur. J. Cancer*, **17**, 449.

THORUD, E., FOSSA, S.D., VAAGE, S. & 4 others (1986). Primary breast cancer: flow cytometric DNA pattern in relation to clinical and histopathological characteristics. *Cancer*, **57**, 808.